# Quantum imaging of the reconfigurable VO_2_ synaptic electronics for neuromorphic computing

**DOI:** 10.1126/sciadv.adg9376

**Published:** 2023-10-04

**Authors:** Ce Feng, Bo-Wen Li, Yang Dong, Xiang-Dong Chen, Yu Zheng, Ze-Hao Wang, Hao-Bin Lin, Wang Jiang, Shao-Chun Zhang, Chong-Wen Zou, Guang-Can Guo, Fang-Wen Sun

**Affiliations:** ^1^ CAS Key Laboratory of Quantum Information, University of Science and Technology of China, Hefei 230026, China.; ^2^ CAS Center for Excellence in Quantum Information and Quantum Physics, University of Science and Technology of China, Hefei 230026, China.; ^3^ National Synchrotron Radiation Laboratory, School of Nuclear Science and Technology, University of Science and Technology of China, Hefei 230029, China.; ^4^ Hefei National Laboratory, University of Science and Technology of China, Hefei 230088, China.

## Abstract

Neuromorphic computing has shown remarkable capabilities in silicon-based artificial intelligence, which can be optimized by using Mott materials for functional synaptic connections. However, the research efforts focus on two-terminal artificial synapses and envisioned the networks controlled by silicon-based circuits, which is difficult to develop and integrate. Here, we propose a dynamic network with laser-controlled conducting filaments based on electric field-induced local insulator-metal transition of vanadium dioxide. Quantum sensing is used to realize conductivity-sensitive imaging of conducting filament. We find that the location of filament formation is manipulated by focused laser, which is applicable to simulate the dynamical synaptic connections between the neurons. The ability to process signals with both long-term and short-term potentiation is further demonstrated with ~60 times on/off ratio while switching the pathways. This study opens the door to the development of dynamic network structures depending on easily controlled conduction pathways, mimicking the biological nervous systems.

## Teaser

Conducting filaments in VO_2_ imaged by quantum sensors is found to be controlled by lasers, helpful for the neuromorphic computing.

## INTRODUCTION

The human brain exhibits impressive talents in cognitive signal processing, analysis, reasoning, and control, depending on hierarchically organized neurons connected by functional synapses (*1*–*4*). Inspired by neurosynaptic frameworks, neuromorphic computing has demonstrated extraordinary capabilities in silicon-based artificial intelligence, for example, supporting AlphaGo to outperform human players at the strategic board game Go (*5*–*7*). The artificial synapses using the functional materials have attracted tremendous interest due to their multilevel resistivity and built-in memory, providing an optimized alternative to silicon-based neuromorphic computing to reduce energy consumption and improve processing efficiency (*8*–*10*). However, the research efforts have focused on two-terminal devices as a complement to silicon-based network structure (*8*–*15*).

**Fig. 1.**
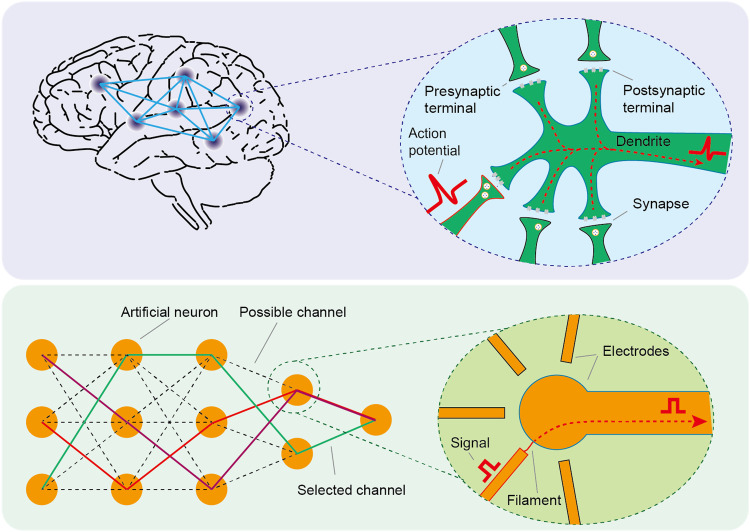
Schematic diagram of the dynamic network structure inspired by the brain. The brain is organized by an interconnected neural network. Action potentials are transmitted through synapses between neurons. Temporal sequential relationships of action potentials can bring about short-term potentiation at active synapses and short-term impressive at other synapses, resulting in an ever-changed neural network. Artificial neurons are constructed by Mott materials, which can be organized hierarchically into dynamic network structures depending on the selection of conducting pathways connected with filaments and multilayer electrodes. Some selected channels are connected as encoded pathways, displayed as lines of different colors. Signal processing and storage in this dynamic network structure depend on the easily controlled encoded pathways that work in similar algorithms to that of the nervous system.

Because of the strongly correlated property, the insulator-metal transition (IMT) in Mott materials induces a huge change in resistivity, and it can be triggered by electric field, enabling applications on artificial synapses (*15*–*20*). Selective phase transition based on the external stimuli manipulates the formation of the conducting filament although its location is still unchangeable (*21*). Using external stimuli with variable positions, the dynamic network can be organized hierarchically by the conducting filaments with controlled locations in Mott materials and multilayer electrodes, similar to biological neurosynaptic frameworks (Fig. 1), which is easily scalable (*22*). The dynamic networks can operate by the competition between the channels in which the nucleation process of metal phase is crucial, similar to the algorithm of neural systems in which the neurons compete with each other to activate and connect to specific pathways encoded by synaptic plasticity (*3*, *4*). In addition, it is reported that quantum sensing can provide a fundamentally different approach to imaging the current-density distribution by detecting the induced stray magnetic fields with high spatial resolution and negligible disturbances, but the imaging of conducting filament in Mott material is still absent (*23*–*25*).

In this study, we propose a dynamic network structure in vanadium dioxide (VO_2_) by controlling the location of filament formation based on the electric field–induced localized IMT. A quantum imaging method for conducting filaments in VO_2_ is creatively developed to directly realize conductivity-sensitive detection with little perturbation and approach the limit of optical spatial resolution of 500 nm. We find that the arbitrary manipulation of filament location can be realized through initializing the IMT by heating a local hotspot with the focused laser, which is applicable to simulate the dynamical synaptic connections between the neurons. The synaptic cooperation and competition, important for the operation of biological nervous system, is investigated in a network structure consisting of several artificial synapses. The conducting filament can be switched arbitrarily to create different synaptic connections with high on/off ratios, allowing the implementation of complex network structures. Thus, the dynamic network is proposed to be hierarchically organized by the artificial synapses, and the conducting pathways can be tuned by controllable filament locations. By investigating the properties of the long-term and short-term potentiation and transmitted signals in the network structure, it demonstrates a promising framework for processing and storing signals based on the encoded dynamic network structures, similar to the operation mechanisms of biological nervous systems.

## RESULTS

### Properties of VO_2_ film and imaging approach

VO_2_ is one of many Mott materials that undergo IMT upon stimulation by temperature, strain, doping, and electric fields (*26*–*34*). Studies have shown that it has the potential to achieve artificial synaptic with plasticity due to negative differential resistance and thermal hysteresis (*33*, *35*, *36*). Because of its special electric field–triggered phase transition features such as the ultrafast response, low-power consumption and high-resistance change rate, VO_2_ provides a nonlinear dynamical response to input signals, as needed to construct neuronal circuit elements. For example, it was reported that the VO_2_-based artificial neurons provide a solution towards large-scale, highly parallel, and energy-efficient in-memory computing systems for deep neural networks (*27*). On the other hand, electric field–induced IMT in a two-terminal device constructed by VO_2_ films can produce the filamentary conducting channel from the original insulation layer. The conducting filament has been imaged on the basis of the distribution of temperature, crystal phase, or optical reflectivity (*16*–*20*). The quantum sensor based on a nitrogen-vacancy (NV) center in diamond enables synchronous measurement of local magnetic field and temperature on VO_2_ film, providing an unusual insight into the field-induced IMT (*25*, *37*). Meanwhile, conducting filaments composed of silver nanowires have been imaged by NV-center ensemble with high spatial resolution (*24*). Compared with the traditional methods (*38*–*41*), this quantum sensing can provide a fundamentally different approach to imaging current-density distribution of conducting filaments in Mott materials, by detecting the induced stray magnetic fields (*23*–*25*). With the application of the lock-in amplifier technique, this method features almost robust, noninvasive, and capable of measuring weak signals with nanoscale resolution. Thus, the quantum sensing technique based on NV centers in diamond is first used to directly image the electric field–induced local phase transition in VO_2_ film and to observe the conducting filaments.

**Fig. 2.**
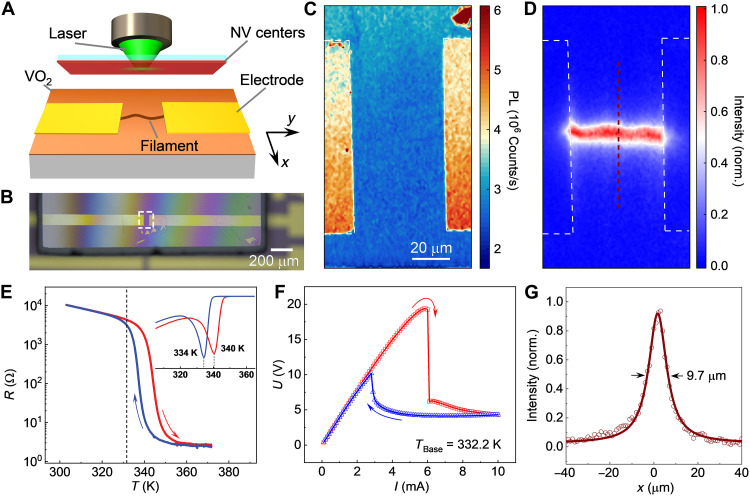
Imaging the conducting filament by diamond NV centers. (**A**) The VO_2_ film (100 nm thickness) growing on c-Al_2_O_3_ is covered by a diamond hosting the NV-center layer implanted about 20 nm below its surface. Electrodes on VO_2_ film are attached to DC current and microwave sources via a bias tee. NV centers are used to detect the stray magnetic fields of microwaves confined in the conducting filament triggered by the DC current. (**B**) A diamond is placed on the VO_2_ device. The gap and width of the Cr/Au electrodes are 50 and 100 μm, respectively. (**C**) Scanning confocal PL image of the NV centers positioned on the device. The PL on the Cr/Au electrodes framed by white dashed lines is substantially higher than that on the VO_2_ film. (**D**) Direct imaging of current-density distribution between electrodes by detecting the normalized intensity of PL modulation of NV centers at 6 mA. Electrodes are framed by white dashed lines. (**E**) Thermally triggered IMT, accompanied by orders of magnitude drop in resistance. The red and blue curves correspond to the heating and cooling branches, respectively. The dashed line shows the base temperature of 332.2 K for the subsequent measurement, with obvious resistance variation induced by thermal hysteresis. The inset is the corresponding differential curve showing the critical temperatures. (**F**) Current-induced IMT. The red and blue curves correspond to the increase and decrease of the current, respectively. (**G**) Intensity profile along the red dashed line in (D) shows that the width of the filament is about 9.7 μm.

The measurement stage consisting of a scanning confocal microscope and a diamond chip hosting a dense layer of shallowly implanted NV centers in micrometer proximity (*42*) to the VO_2_ film (Fig. 2, A and B, and section S1). The filament can be triggered by a DC current in the two-terminal device implemented by VO_2_ film. High-frequency signals, such as resonant microwaves used to pump the electron spin resonance of NV centers (*24*), can be delivered to the filament through a bias tee. Figure 2C shows confocal photoluminescence (PL) image of the NV centers placed on a VO_2_ device with a resolution of 500 nm (fig. S2), where the PL on the electrodes is substantially higher than that on bare VO_2_ film caused by the metal-enhanced reflectivity. PL is the main signal measured in NV center–based quantum sensing, which is spin quantum state dependent. A resonant microwave at 2.87 GHz in the absence of an external magnetic field pumps the quantum state of NV centers and subsequently changes the PL (*43*, *44*). The contrast of the optically detected magnetic resonance signal increases with higher microwave power (fig. S3). In this work, a lock-in amplifier is used to improve the signal-to-noise ratio by modulating the microwave power (*45*). As shown in Fig. 2D, the intensity of the modulated PL shows the distribution of the stray magnetic field caused by the microwaves confined in the filament. Figure 2E shows the thermally triggered IMT in the VO_2_ device, accompanied by orders of magnitude decrease in resistance. The insert shows the critical temperatures of about 340 and 334 K for the heating and cooling processes, respectively. The difference between the heating and cooling branches exhibits thermal hysteresis, which facilitates the implementation of the built-in memory. The crystal quality and phase transition temperature of epitaxial VO_2_ film are also characterized by the scanning transmission electron microscope (STEM) and Raman spectroscopy (figs. S4 and S5). The base temperature is stabilized at a constant of 332.2 K in the subsequent measurements, which is marked with the dashed line in Fig. 2E and exhibits obvious resistance variation induced by thermal hysteresis. The voltage between electrodes in response to the gradually varying DC current is shown in Fig. 2F. The sharp drop in voltage at the critical current suggests that a conducting filament stretching between the ends of the electrodes is formed in the VO_2_ film, as shown in Fig. 2D for a 6-mA current. The full width at half maximum of the cross-section profile for the filament is about 9.7 μm, as shown in Fig. 2G, revealing the comparable or better spatial resolution in comparison with previous studies of VO_2_ film (*16*–*19*). According to the analysis based on three-dimensional (3D) finite element simulations (*21*), the distance between the NV-center layer and VO_2_ film is about 1 to 2 μm in this case (fig. S7). The spatial resolution can be further optimized by employing optical super-resolution microscopy techniques and by positioning the NV-center layer in closer proximity to the film under study (*24*).

### Controllable filament formation location by laser heating

In contrast to the spontaneous conducting pathways in metal nanowire networks, including the silver nanowire networks (*46*), conducting filaments in the VO_2_ film are the result of local temperature redistribution, and thus, by controlling local heat flow, the encoded network structure organized by Mott materials can be dynamic and reproducible. It is shown that the filament formation is triggered by nucleation at hotspots with a subsequent expansion above the threshold current in a VO_2_-based two-terminal device. The localized nucleation with reduced resistivity can concentrate the current flow and induce Joule heating, bringing about a positive feedback loop (*16*, *17*). Therefore, the field-induced IMT process manifests itself as a discontinuous drop, as shown in Fig. 2F, accompanied by the convergence of the current into a filament. The hotspot is the initial point in space for field-induced IMT and determines the filament location. However, the spontaneous hotspots depend on the distribution of defects and initial current density in the VO_2_ layer, which are unchangeable and bring about an immovable filament location.

**Fig. 3.**
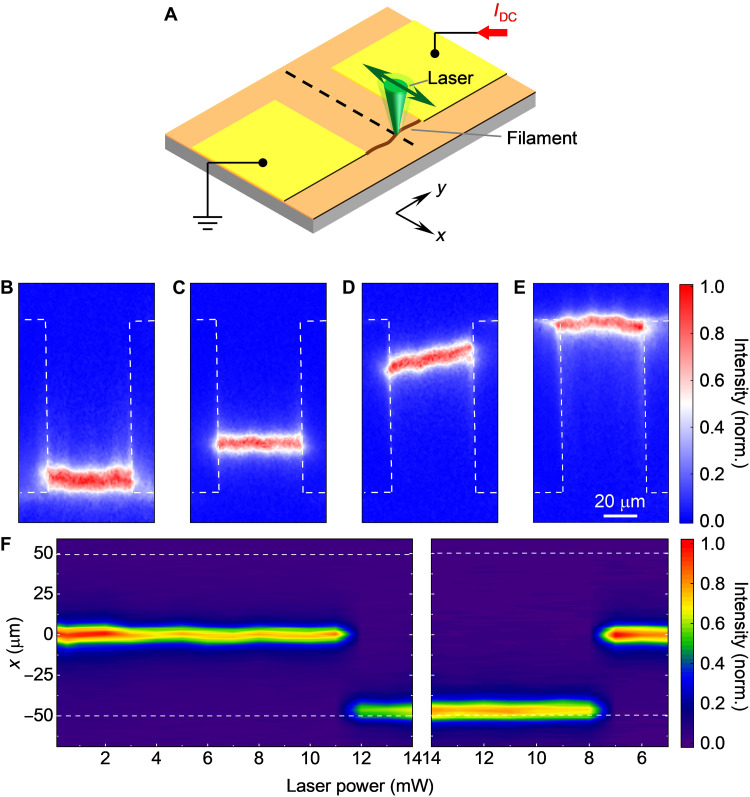
Control of filament location by focused laser. (**A**) Operating procedure for controlling the filament location. The laser is focused on the selected point of the dashed line on the VO_2_ device with adjustable power, and the filament is triggered by a DC current before being detected by NV centers. The green arrow indicates the movement direction of the focused laser used for localized heating. (**B** to **E**) Imaging of normalized PL modulation intensity for the cases with laser focused at *x* = −50, −25, 25, and 50 μm, respectively. The power of the laser is set to 14 mW. The filament locations are successfully shifted to the positions of the focused laser. Electrodes are framed by white dashed lines. (**F**) Normalized PL modulation intensity is scanned in the direction perpendicular to the current, along the dashed line shown in (A), in response to different laser powers. The laser heating position is fixed at *x* = −50 μm in this case. The white dashed lines mark the boundaries of the electrode pair in the VO_2_ device. It shows that the filament location is shifted to the laser position when the laser power is increased to 12 mW (left) and returns to the spontaneous location when decreased to 7.5 mW (right).

Here, we create an arbitrarily controllable artificial hotspot by focusing the laser onto VO_2_ film so that the formation location of conducting filament can be manipulated. Figure 3A shows the operation process, where the focused laser is used to heat a selected point between the electrodes along the dashed line, and the DC current is sequentially increased above the threshold to trigger the filament. The diameter of the spot is less than 500 nm (fig. S2). The laser power is then adjusted to a lower value for detecting the location of the filament. As shown in Fig. 3B, The PL modulation intensity indicates that the filament is successfully shifted to the laser position, while the laser-heating position is at *x* = −50 μm with a high power of 14 mW. The cases of different laser positions exhibit similar controllable filament locations, as shown in Fig. 3 (C to E), so the position of focused laser–induced artificial hotspots can be moved to control the location of the conducting filament. Another parameter that affects the filament location is the power of the heating laser. When the focused laser is at a fixed position *x* = −50 μm, the PL modulation intensity is scanned along the dashed line shown in Fig. 3A for quick detection of the filament location. Figure 3F illustrates that while increasing the laser power, the filament location switches from *x* = 0 to −50 μm at 12 mW but back to *x* = 0 μm at 7.5 mW. Another device confirms the hysteresis of filament location depending on laser power (figs. S10 and S11). A sufficient interval of several minutes is left to avoid the effect of the adjacent Joule heat dissipation between tests in the experiment (fig. S12). Thus, the hysteresis of the filament location depending on laser power is the result of a nonvolatile feature that can be attributed to the thermal hysteresis of the VO_2_ film. 3D finite element software is used to simulate the moveable filament caused by laser heating, which also demonstrates the feasibility of controllable filament location (fig. S8).

### The synaptic cooperation and competition among the VO_2_-based devices

**Fig. 4.**
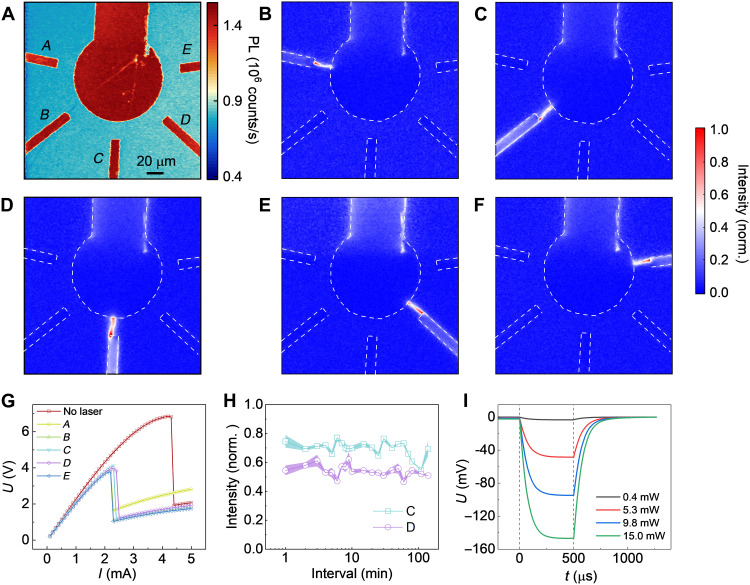
The focused laser–controlled synaptic cooperation among VO_2_-based devices. (**A**) Confocal PL image of the NV centers in proximity to a network structure consisting of five artificial synapses constructed by a VO_2_ film. The common terminal is a rounded electrode, mimicking a biological dendrite, attached to DC current and microwave sources via a bias tee. The other five electrodes labeled *A* to *E* are attached to the ground, similar to presynaptic terminals. (**B** to **F**) Normalized PL modulation intensity imaging while focusing the laser to the middle of channels *A*, *B*, *C*, *D*, and *E*, respectively, and triggering the filament by a DC current. The filament location can be switched among the five channels. Electrodes are framed by white dashed lines. (**G**) The current-voltage curve when the laser is focused to the middle of each channel with obviously reduced critical current. (**H**) The PL modulation intensity is measured on channel *C* or *D*, when the corresponding channel is individually triggered by DC current after the initial selection of laser and different intervals. The intensity should be larger than 0.2 when the measured channel is turn on. Thus, there is a long-term potentiation for hours on channel selection in the network structure. (**I**) The variation of voltage in channel *C* depending on the pulsed laser focused on the conducting filament. The pulsed laser is turned on at 0 μs and off at 500 μs with a DC current of 6 mA to stabilize the conducting filament. The saturation time is about 300 μs. The DC voltage component is removed in the measurement. Thus, there is a short-term potentiation induced by pulsed lasers with different powers.

On the basis of the controllable conducting filament location, we propose an artificial dynamic network structure scheme for mimicking the functions of neural synapses in biological systems, in which the synaptic cooperation and competition is important for the operation and stabilization of the network structure. As shown in Fig. 4A, the basis is a network structure composed of functional synapses constructed by VO_2_ film. The common terminal is a round electrode that mimics a biological dendrite and is attached to a DC current and microwave source via a bias tee. Other electrodes, resembling presynaptic terminals, are attached to the ground. The conducting filament is formed as the DC current gradually increases above the threshold, instead of the initial metastable state. The spontaneous filament location relays on the defect-induced hotspots so that only one channel with the most defects can be triggered. Nevertheless, considering the excellent quality of the VO_2_ film, we can use focused-laser heating to generate a stronger hotspot and rearrange the location of filament formation. We imaged the intensity of PL modulation as shown in Fig. 4 (B to F), after focusing the laser onto the bare VO_2_ film in the middle of each channel with a power of 10 mW, accompanied by a decrease in the critical current as shown in Fig. 4G. The results show that the hotspot generated by laser heating can effectively control the location of the conducting filament, which is also verified by simulation (fig. S9). It is worth mentioning that the filament-forming channel is exclusive, that is, the accumulation of current to one filament prevents the formation of filaments on other channels, similar to the short-term depression caused by action potentials in biological neurons (*6*).

The size of device is important for integration, which affects the energy consumption and the complexity of the network. While the size of device is smaller than the spot diameter of the laser, the selection between adjacent synapses would be disabled due to indistinguishable laser-heating effect. In experiment, we deduce that stable control of filament location could be achieved with the spacing limitation of 1 μm between adjacent artificial synapses (fig. S13), which is attributed to the localized temperature distribution induced by laser heating and the relatively high thermal conductivity of the substrate.

### Built-in memory and signal processing depending on dynamic network

Figure 2E illustrates the effect of thermal hysteresis on the resistivity of VO_2_ film, which can bring plasticity to artificial synapses and generate nonvolatile memory. Channel *C* in Fig. 4A is chosen to study the memory of the filament-formation location. We find that when a channel is selected by a laser and triggered by a DC current, the network is still affected by such initial state for quite a long time after removing the stimuli including the DC current and laser, which is manifest as that the DC current could retrigger this channel without the laser. The on/off of the channel can be determined by the PL modulation intensity on corresponding channel, which should be higher than 0.2 while the channel is turned on. Figure 4H shows that the channel *C* can be selected to be triggered without laser after the initial selection by laser, so that channel *C* exhibits long-term potentiation for up to several hours, which is also verified on channel *D*. The thermal hysteresis should be responsible for long-term memory. In terms of current density distribution, the channel with lower resistance when retriggering has a clear competitive advantage over other channels, so the filament tends to form on the same channel (fig. S12). The pulsed laser–induced short-time potentiation is also found by oscilloscope monitoring of the voltage between electrodes after the filament is stabilized by a 6-mA DC current. Figure 4I shows the apparent response to a pulsed laser with a saturation time of approximately 300 μs. Laser power is a key parameter for the synaptic weight of network structure in short-term potentiation. The network structure can be organized hierarchically into a dynamic neuromorphic structure (Fig. 1), aided by laser-controlled filament location with nonvolatile memory. The connection pathways in such neuromorphic structure can be easily controlled and have both long-term and short-term potentiation, providing built-in memory according to encoded conduction pathways similar to those of biological nervous systems.

**Fig. 5.**
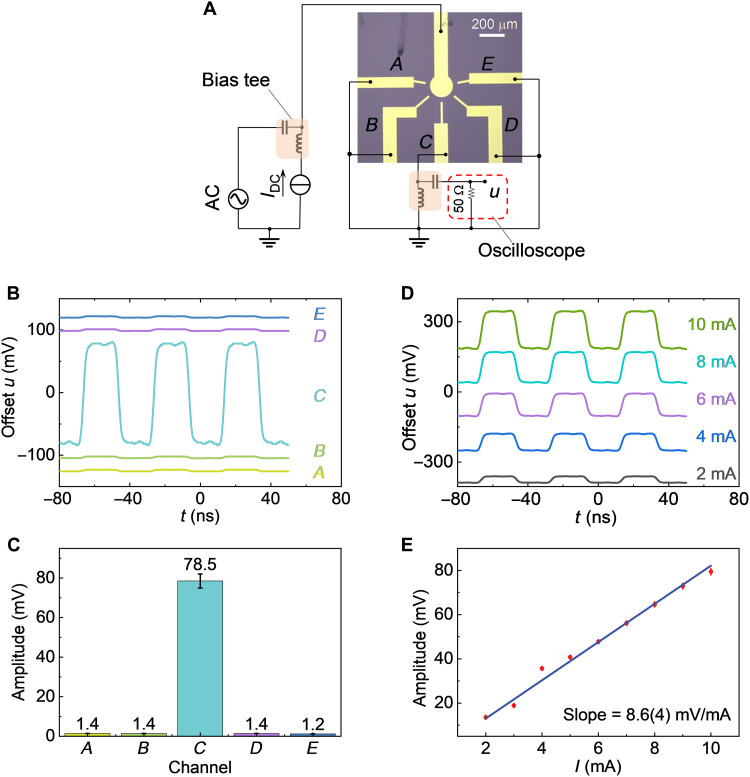
Adjustable signal transmission based on the synaptic cooperation. (**A**) Method of capturing the signals transmitted through the network structure consisting of artificial synapses. A square wave with a frequency of 25 MHz and an amplitude of 0.5 V is connected to the common electrode through a bias tee as a signal source. Another bias tee is used to connect an oscilloscope with a 50 ohms internal resistance in series between channel *C* and the ground to monitor the transmitted AC signal. (**B**) Transmitted signal of channel *C* when a filament is formed in each channel selected by the focused laser and triggered by a 10-mA current. (**C**) The amplitude of the transmitted signal as the filament is formed in each channel. When the filament is formed in channel *C*, its amplitude is about 60 times higher than the other channels. (**D**) The transmitted signal of channel *C* is controlled by DC current as the filament is formed in channel *C*. (**E**) The proportional relationship between the amplitude of the transmitted AC signal and the DC current within the tolerable range. The slope is 8.6(4) mV/mA.

Signal processing in the synapse is also important for the realization of artificial dynamic network structures. Instead of the resonant microwave used in previous experiments, a 25-MHz square wave is attached to the common electrode through a bias tee as a signal source. Another bias tee is connected to an oscilloscope to monitor the transmitted signal with 50 ohms internal resistance in series between channel *C* and the ground, as shown in Fig. 5A. The transmission signal of channel *C* changes markedly, as shown in Fig. 5B, while the conducting filament is selected by the laser and triggered by the DC current in each channel. The transmission signal is approximately 60 times stronger when channel *C* is triggered, as shown in Fig. 5C. Thus, the transmitted signal through each channel is competitive and has a high on/off ratio of about 60 times, which is crucial for the implementation of complex network systems, due to the reduction of cross-talk and noise (*15*, *22*). In addition, the DC current stabilizing the conducting filament has a substantial effect on the resistance of the conducting channel, so the transmitted signal can be manipulated, as shown in Fig. 5D. Figure 5E illustrates the proportional relationship between the amplitude of the transmitted signal and DC current within a tolerable scale. In this way, the network structure exhibits the function of signal processing through changeable filament-formation location and tunable channel resistance, paving the way for neuromorphic computations that depend on the dynamic network structure.

## DISCUSSION

In conclusion, on the basis of the focused laser–controlled filament location, we propose a scheme to create a dynamic neuromorphic structure organized hierarchically by VO_2_-based artificial synapses. A diamond NV center–based quantum sensing method with little perturbation and fairly high resolution is applied for conductivity-sensitive imaging of filaments in VO_2_ films and the basic properties of network structure. Variable signal processing and long-term and short-term potentiation are investigated to construct the encoded dynamic network structure with an algorithm similar to that of multichannel signal processing and storage in the biological nervous system. The high on/off ratio suggests the ability to implement complex network systems. The stimulus to select the channel in our work is a focused laser that should be extended in the future to an arbitrary variable light field to enable the parallel manipulation of a large number of artificial synaptic connections. Collectively, the studies should contribute to the development of hardware neural network systems with brain-inspired algorithms and facilitate the application of neuromorphic computing.

## MATERIALS AND METHODS

### Sample fabrication

VO_2_ films with a thickness of 100 nm are grown on c-Al_2_O_3_ substrates by a radio frequency–plasma–assisted oxide molecular beam epitaxy instrument working at a base pressure lower than 3 × 10^−9^ torr. During the deposition process, the substrate temperature is maintained at 823 K and the growth pressure is kept at 3 × 10^−5^ torr. Reflection high-energy electron diffraction is used to monitor the entire growth process. For electrical transport tests, chromium/gold (10 nm/100 nm) electrodes are fabricated on top of the VO_2_ film by electron-beam lithography (PMMA A4 resist) and thermal evaporation.

The diamond used in this work is a chemical vapor deposition–grown, electronic-grade type IIa crystal (Element 6). The crystal is cleaned with tri-acid (mixture of nitric, sulfuric, and perchloric acid, 1:1:1) and implanted with ^15^N ions at 15 keV with a dose of 1 × 10^13^ ions/cm^2^ before annealing at 800°C for 3 hours at approximately 1 × 10^−4^ Pa and sequentially cleaning with tri-acid to remove the graphitized layers from the surface. The density of NV centers is estimated to be about 2 × 10^10^/cm^2^ and the NV layer is about 20 nm under the surface.

### Measurement setup

The optical setup used for all the imaging measurements is a homemade scanning confocal microscope. A 532-nm laser (MLL-III-532 nm, New Industries Optoelectronics) is used to optically excite the diamond NV centers. The laser is focused on a spot with a diameter of 500 nm by a Leica 63×, numerical aperture of 0.70 correction collar objective. The PL of NV centers collected by the same objective is detected by a single-photon counting module (SPCM-AQRH-15-FC, Excelitas) and separated from excitation light by a Semrock dichroic mirror and long-pass filter (637-nm cutoff) and spatially filtered by a pinhole (50 μm, Thorlabs). The microwave signal used to drive the NV centers is provided by a Rohde & Schwarz microwave generator (SGS100A), and the microwave pulses are controlled by a microwave switch (ZASWA-2-50DR, MiniCircuits). The signals from SPCM are replicated by a field-programmable gate array board and connected to a lock-in amplifier (SR830, Stanford Research Systems) and National Instruments data acquisition card to detect PL intensity and the intensity of PL modulation by microwave power, respectively. A SpinCore programmable pulse generator (PulseBlasterESR-PRO 500) is used to control the microwave switch and provide a reference signal for the lock-in amplifier. The position of the focused laser is changed by piezo-stage (P-562.3CD, Physik Instrument). The base temperature is controlled by a temperature controller (TC200, Thorlabs). The electrical platform includes a DC current source and a nanovoltmeter (6221 and 2182, Keithley) through a bias tee (ZFBT-4R2GW+, MiniCircuits). Measurements of the transmitted signal are made using an AC signal source (SDG6032X-E, Siglent) and an oscilloscope (TBS1102B, Tektronix). High-angle annular darkfield STEM images are acquired by a JEOL JEM-ARM200F microscope incorporated with a spherical aberration correction system operating at 200 kV with a resolution limit of 0.08 nm. Variable-temperature Raman spectra are recorded by an integrated laser Raman system (LabRAM HR, Horiba) that has a variable-temperature sample stage and uses a 532-nm He-Ne laser as the excitation source.

### 3D finite element simulation

The 3D finite element model of our devices uses COMSOL Multiphysics, which self-consistently considers electrical and thermal effects. We apply the interface on electric current and heat transfer in the software for steady-state analysis. The voltage and current distribution in the device are calculated by the electrical model, while the thermal model determines the temperature distribution.

∇⋅[σ(T)∇V]=0 (1)

∇⋅(k∇T)+J⋅E=0 (2)

Equation 1 is used for the analysis of the voltage and current distribution according to Ohm’s law and the conservation of charge, while Eq. 2 allows the derivation of the local temperature according to Fourier’s law, energy conservation, and Joule’s law. The electrical and thermal analysis is coupled by Joule heat *J* ⋅ *E* and temperature-dependent local conductivity σ (*T*) of VO_2_ layer.
