# Supplementary material for: Quantum imaging of the reconfigurable VO2 synaptic electronics for neuromorphic computing
Source: Sci Adv. 2023 Oct 4;9(40):eadg9376. doi: 10.1126/sciadv.adg9376 (PMC10550222; doi:10.1126/sciadv.adg9376)
Supplement: Supplementary file 1 — Supplementary Text Figs. S1 to S13 Table S1 [file sciadv.adg9376_sm.pdf]

Science Advances
AAAS

Supplementary Materials for

Quantum imaging of the reconfigurable VO_2 synaptic electronics for neuromorphic computing

Ce Feng *et al.*

Corresponding author: Fang-Wen Sun, fwsun@ustc.edu.cn; Chong-Wen Zou, czou@ustc.edu.cn

Sci. Adv. **9**, eadg9376 (2023)
DOI: 10.1126/sciadv.adg9376

This PDF file includes:

Supplementary Text
Figs. S1 to S13
Table S1

Supplementary Text

S1. Experimental platform and calibration based on NV centers

The NV centers exhibit quantum properties similar to traditional atomic systems for sensing application, but the nature of point defects for NV centers suits the spatially resolved sensing technique. As shown in Fig. S3A, the NV center consists of a nitrogen atom and an adjacent vacancy, replacing the carbon atoms in the diamond, and the symmetry axis is constrained along one of four <111> crystallographic direction in diamond. It should be mentioned that the negatively charged NV center is applied in our experiments while the NV centers exist in three charge states according to the observation. The basic properties of the negatively charged NV center (called NV center for simplification) are mainly shown in this section, and the imaging method relies on the optically detected magnetic resonance (ODMR) effect.

The NV center is a spin triplet in both electronic ground state ($^3A_2$) and excited state (^3E). The optical transitions between these levels are primarily spin conserving, except for the non-radiative spin-state-dependent relaxation mechanism through an intersystem crossing to the spin singlet (^1A) and a decay to $m_S = 0$ spin state in electronic ground state. The spin-selective nature of transition process generates the capacity for the spin state to initialize to $m_S = 0$ under optical pumping and the detection of spin states depending on the photo-luminescence (PL) signal. In particular, the PL is larger when $m_S = 0$ than when $m_S = \pm 1$, induced by the higher probability of non-radiative relaxation for $m_S = \pm 1$. In conclusion, the spin state in the ground state can be detected by PL intensity. The transitions of spin states in $^3A_2$ can be induced by applying a microwave magnetic field with a specific frequency corresponding to the electron spin resonance (ESR) process, shown as the PL reduction in Fig. S3C. While the PL signal could be disturbed by perturbations such as power jitter of laser, the lock-in application is employed in our experiments to highlight the effect of microwaves. As shown in Fig. S1, the microwave is turned on and off to modulate the microwave intensity, and the corresponding PL intensity is tested and de-modulated to obtain the dependence of the PL-modulation intensity on the power and frequency of microwave. The results for the NV centers positioned on the conducting filament in VO_2 film, which is fed with microwaves and establish the magnetic field on the film, are exhibited in Fig. S3E. The PL-modulation intensity depending on the microwave, shown in Fig. S3F, creates the basic for the detection for the microwave-magnetic-field distribution to achieve the conducting-filament imaging.

In VO_2 device, the formation location of conducting filament is controlled by the laser or defects in VO_2 film and generated by DC current, and thereafter the microwave with resonant frequency is delivered into the conducting filament thought the bias-tee. The spatially resolved detection of PL-modulation intensity of NV centers placed on the VO_2 device is conducted, which reflects the distribution of microwave magnetic field and the current. Accordingly, the imaging of the conducting filament can be realized based on the distribution of current density.

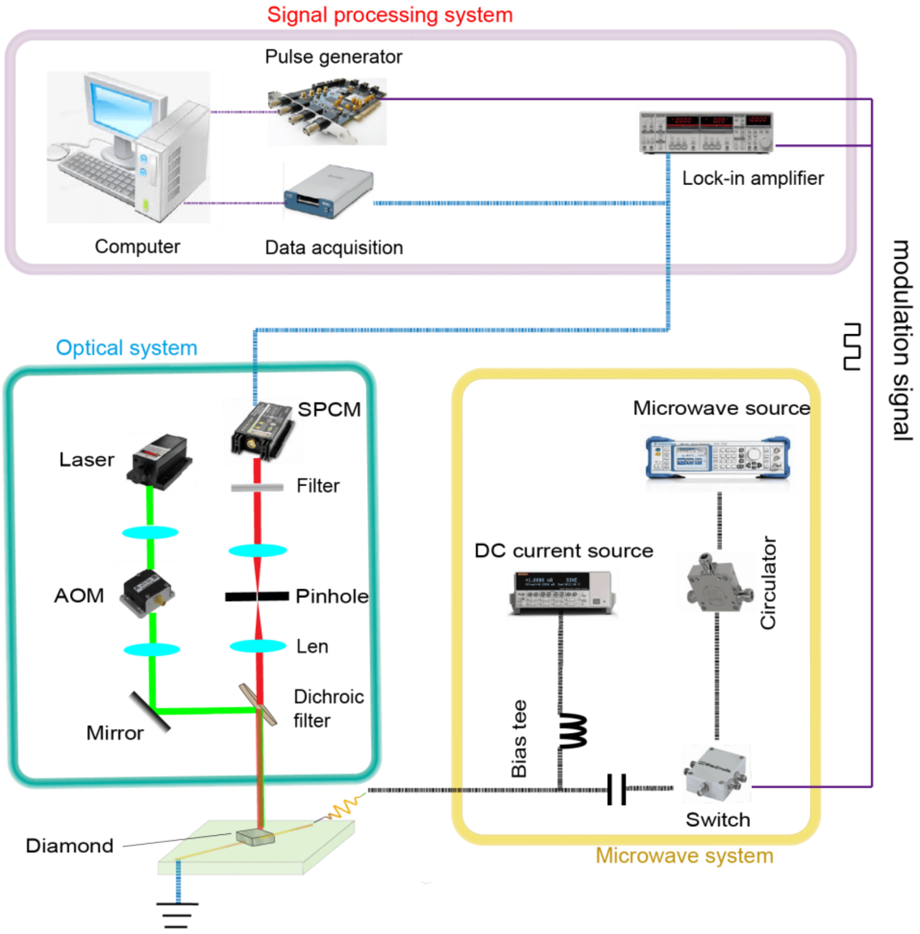

Fig. S1. The experimental platform. The platform consists of an optical system, a microwave system and a signal processing system, as well as a piezoelectric translation stage and a heating stage below the sample. The laser with a wavelength of 532 nm is focused on the NV centers by an objective through an acoustic-optical modulator (AOM) and a dichroic filter. The PL of NV centers is collected by the same objective and detected by a single-photon counting module (SPCM) through a dichroic filter, a long-pass filter, and a spin-hole to improve spatial resolution in the depth direction of the sample. The microwave is attached through radio-frequency (RF) port of a bias tee, a circulator, and a switch, and the DC current source is attached through the DC port. The pulse generator provides the transistor-transistor logic (TTL) signal, a square wave with 25 kHz, to the microwave switch and the reference signal to lock-in amplifier. The PL of NV centers is modulated by the microwave power, and the de-modulated intensity by the lock-in amplifier is acquired through data acquisition and a LabView program in the computer.

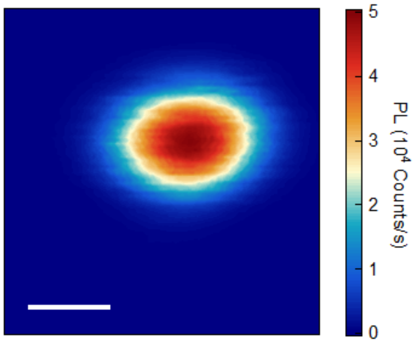

Fig. S2. The PL-intensity distribution of the NV centers that is confined in a disk with a diameter of 100 nm. The focused laser spot and the spatial resolution of the fluorescence microscope is about 500 nm. The scaler bar is 500 nm.

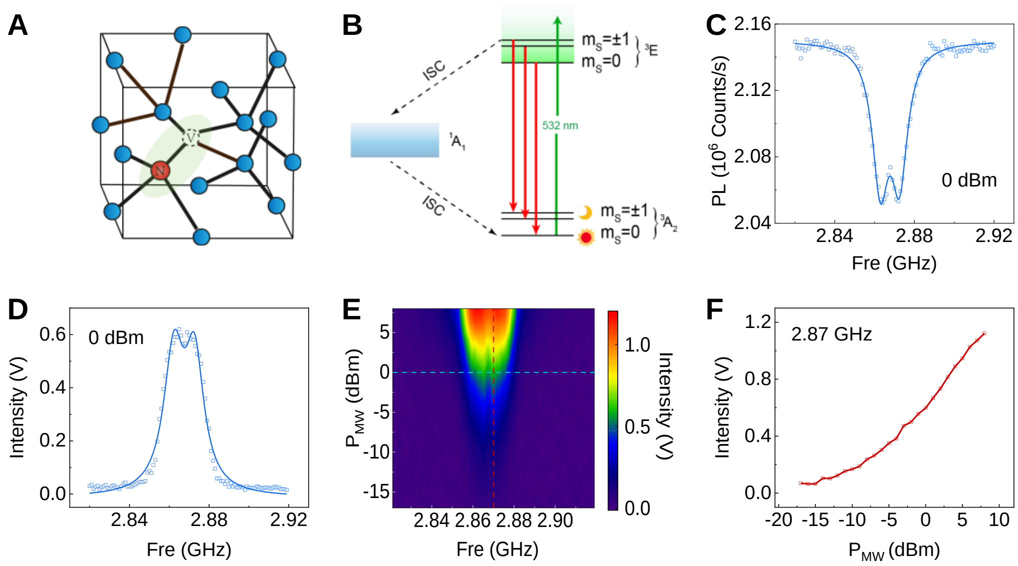

Fig. S3. Properties of NV centers. (**A**) The NV center consists of a nitrogen atom and an adjacent vacancy, replacing the carbon atoms in the diamond. (**B**) The state of NV center can be initialized by the 532-nm laser to $m_S = 0$, which exhibits a higher PL than $m_S = \pm1$. Resonant microwaves can bring about ESR and induce lower PL. (**C**) The ODMR signal measured by data acquisition at a power of 0 dBm responds to the microwave frequency. (**D**) The ODMR signal measured with a lock-in amplifier by modulating the microwave power. (**E**) Intensity of the modulated PL measured with different microwave powers and frequencies. (**F**) The intensity of the modulated PL with a resonant microwave frequency of 2.87 GHz is positively correlated with the microwave power.

S2. The crystal structure and phase characterization of VO_2 layer

To further confirm the quality and in-plane orientations of the crystal VO_2/c-sapphire thin-film heterostructure, high-angle annular dark field images (HAADF) and selected area electron diffraction (SAED) patterns are performed with scanning transmission electron microscope (STEM). Figure S4A represents the low-magnification HAADF image along the [10-10] sapphire zone axis. The related indexed electron diffraction pattern in Fig. S4B illustrates both (001) and

(100) in-plane orientations, confirming the epitaxial growth of VO_2 (020) on the sapphire crystal substrate as sapphire (01-10) // m-VO_2(001) and sapphire (01-10) // m-VO_2(100), respectively. The high-resolution HAADF images show the alternate stacking of bright and dark regions along the substrate surface in the VO_2 layer, as shown by circles of different colors in Fig. S4C, D. The alternating stacking and uniform lattice spacing confirm the excellent crystallinity and epitaxial relationship in Fig. S4B. A detailed investigation of the bright and dark regions is shown in Fig. S4E, F, respectively, where Zhou et al. have indexed the image of Fig. S4E as the [100] direction of VO_2 and Fig. S4F as VO_2[001] or VO_2[120] (*47*). According to STEM results, it can be observed that there are some dislocations and grain boundaries in the films, as shown in Fig. S4C, D, which is due to the growth of the epitaxial film and the unique crystal structure symmetry of VO_2. While on the one hand from the atomic resolved STEM-HAADF images, the excellent crystal quality of VO_2 epitaxial films is confirmed.

The Raman spectrums tested at different temperatures show that the epitaxial VO_2 film has three distinct V-O vibration peaks at room temperature of 192 cm^{-1}, 223 cm^{-1} and 613 cm^{-1}, which proves that the pristine VO_2 layer sandwiched in Au/Cr/VO_2/Al_2O_3 neural network device is a standard insulating monoclinic phase. As the experimental temperature increases, the VO_2 layer exhibits different degrees of IMT, which causes the intensity of all Raman peaks belonging to A_g and B_g vibration modes to gradually decrease as well. When the temperature exceeds the critical phase-transition temperature of VO_2, all the Raman peaks disappear completely, as shown in Fig. S5. These results indicate that the VO_2 film undergoes an IMT process from monoclinic to tetragonal.

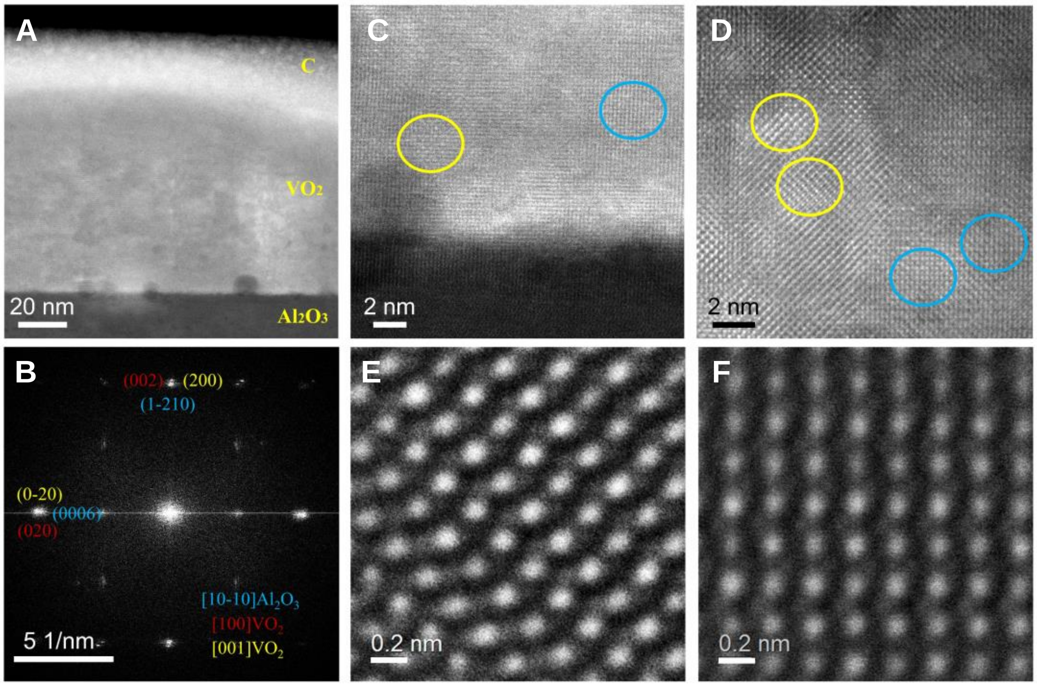

Fig. S4. Crystal structure characterization of epitaxial VO_2 film. (**A**) Low-magnification HAADF image taken by STEM showing columnar growth in both orientations along the axis of the [10-10] sapphire region. (**B**) Diffraction pattern belonging to two crystallites of VO_2 along [001] and [100] and [10-10] substrate. (**C**) HAADF image taken from the interface between VO_2 and sapphire substrate. The dark region represents the lattice phase of sapphire and two sets of VO_2 domains (marked with blue or yellow circles) can be observed at the interface. (**D**) HAADF-

STEM image showing that the epitaxial sample contains two sets of VO_2 domains. A colored circle indicates the corresponding set of domains. (**E**) STEM-HAADF high-resolution image of the bright region in (D) (yellow circles). (**F**) HAADF image of the dark region in (D) (blue circles).

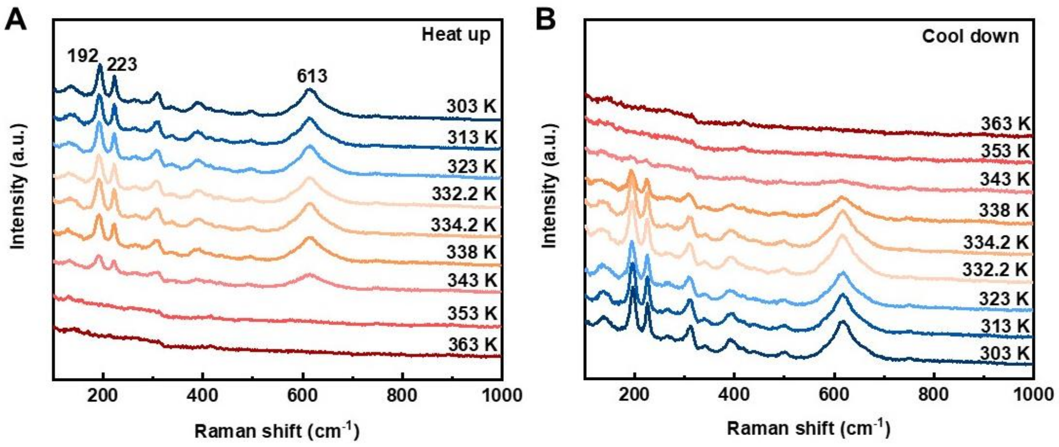

Fig. S5. Variable-temperature Raman spectra of the epitaxial VO_2 film. (**A**) Increasing temperatures. (**B**) Decreasing temperatures.

S3. Three-dimensional finite element simulations on the conducting filament in VO_2 layer

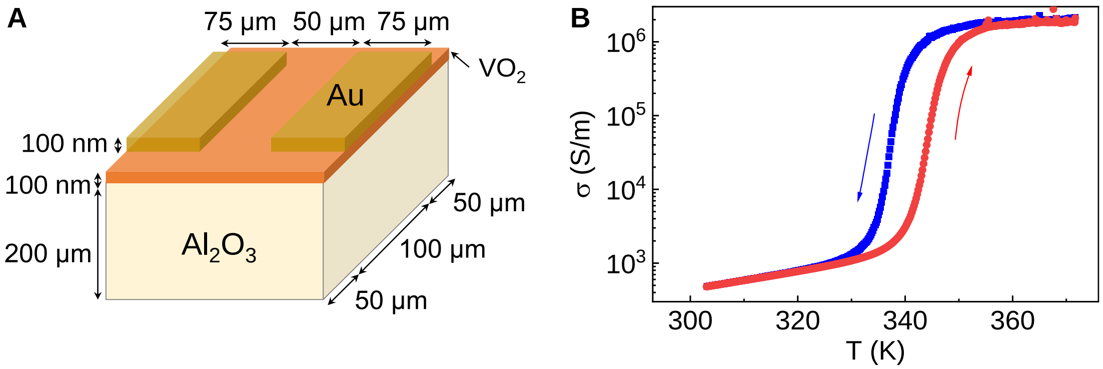

Fig. S6. Parameters imported into the simulation. (**A**) Device geometry used to simulate the experimental device in the main text. (**B**) $\sigma(T)$ data for VO_2 layer is calculated from the temperature-dependent resistance shown in Fig. 2E and the distribution of weak current flow.

Figure S6A shows the device geometry used for the finite element simulation, where the volume of the Al_2O_3 substrate is set to 200 μm × 200 μm × 200 μm to satisfy the complete attenuation of the current-induced Joule heat at the interface. The bottom surface of substrate mounted on the heating stage in the experiment is set to an isothermal surface of 332 K. The conductivity of the VO_2 layer is calculated as a function of temperature from the experimental $R(T)$ data. $\sigma(T)$ data is imported as a table of temperature and conductivity in 0.2 K increments shown in Fig. S6B, which is used as the nearest-neighbor interpolation type. The default linear interpolation allows for a better fit of the $\sigma(T)$ curve to the experimental data, but small temperature changes can lead to

large changes in resistivity near the critical temperature of the VO_2 layer, driving the simulation to fail to converge and providing illogical results. Table S1 lists other material parameters used in our simulation.

Table S1. Materials properties used in simulation.

	σ [S/m]	K [W m^{-1} K^{-1}]
Al_2O_3	10^{-20}	34.5
VO_2	function of T(x, y, z) spanning 460 to 2.1×10^6	5
Au	4.4×10^7	317

We have completed the simulation on conducting filament using a direct iterative approach. The initial condition for the next current is the solution of the previous simulation step. The damping factor for the iterative temperature distribution calculation should be as low as 0.05 to avoid the oscillations of temperature distribution in each iteration. For each simulation, only one branch of the $\sigma(T)$ curve (heating or cooling branch) is applied at a time. In general, the heating branch is used for increasing-current simulations, while the cooling branch is used for simulations with decreasing currents. Thus, there is a significant difference in the current-density distribution at 6 mA for both increasing and decreasing cases, as shown in Fig. S7A, B. The conducting filament can be maintained when the current is reduced from 12 mA above the threshold. The simulated current-voltage curves are similar to the experimental data, as shown in Fig. S7C, assisted by the thermal hysteresis on resistivity and the different initial states for simulation. The NV-center layer is placed on the VO_2 layer at a distance of micrometers, as shown in Fig. S7D, where the current-induced stray magnetic field can be calculated through integration according to Oersted's law. Figure S7E shows a cross-sectional plot of the current density along the dashed line in Fig. S7B, so that the stray magnetic field induced by the current flow in conducting filament can be derived based on Equations (1-3),

$$\vec{H}(x_0, y_0) = H_x(x_0, y_0)\hat{e}_x + H_y(x_0, y_0)\hat{e}_y, \tag{1}$$

$$H_x(x_0, y_0) = \frac{y_0 t}{2\pi}\int \frac{J(x)dx}{(x_0 - x)^2 + y_0^2}, \tag{2}$$

$$H_y(x_0, y_0) = -\frac{t}{2\pi}\int \frac{J(x)(x_0 - x)dx}{(x_0 - x)^2 + y_0^2}, \tag{3}$$

where t is the thickness of VO_2 layer. Figure S7F demonstrates the normalized magnetic-field intensity when setting the distance of NV-center layer and the VO_2 layer to 1-4 μm, respectively. The blue dots show the normalized intensity of modulated PL in the measurement, which is positively correlated with the microwave magnetic field. Thus, in this case, the distance of the NV-center layer and the VO_2 layer is about 1-2 μm, similar to the results of another work (*42*).

Based on the previous simulation results, the focused laser-controlled filament location is also studied by finite element simulation as shown in Fig. S8. To reduce the degrees of freedom of the simulation, we substitute the effect of laser heating by variable-power heat flow to a specific region of 1-μm diameter disc at x = 50 μm. When increasing power of the focused laser, which is the

power of local heat flow, the filament location can be shifted to the position of laser heating with a critical power of 6 mW, which is smaller than the experimental value of 12 mW due to the photo-thermal conversion efficiency.

The laser-controlled channel switching between artificial synapses of VO_2 film is simulated as shown in Fig. S9. The geometry of the device is similar to that of the experimental device, with channels labeled A, B, C, D, and E, respectively. When the laser is absent, the current density is uniformly distributed in each channel at 10 mA and the conducting filament forms in channel C at 13 mA, due to the small asymmetry in building the model. When the laser is applied in the middle of channel A, the filament forms in the corresponding channel with a smaller critical current. The filamentary conducting pathway can be maintained in channel A while removing the laser. The other channels show the similar effects, so the local heat flow induced by focused laser can control the filament location based on the simulation of our artificial neuron.

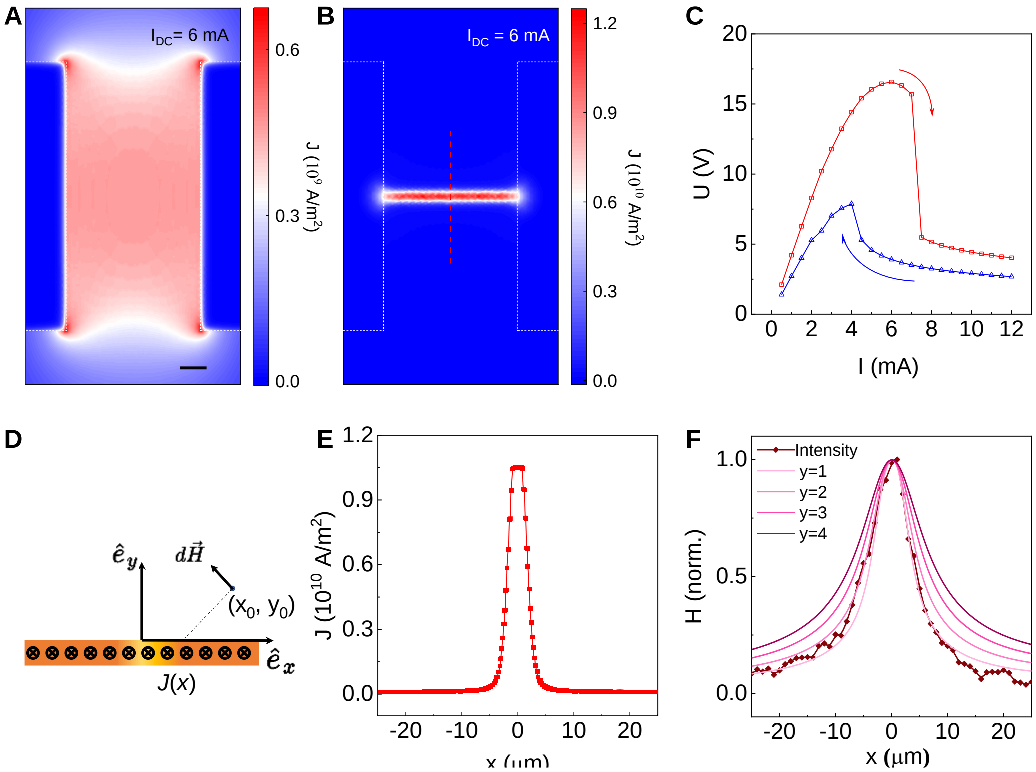

Fig. S7. The simulation on spontaneous current-density distribution and stray magnetic field. (**A**) Current density distribution as the current increases to 6 mA below the threshold. The current density is dispersed between the electrodes. The base temperature in the simulation is 332 K and the scale bar is 10 μm. (**B**) The conducting filament forms as the current decreases from 12 mA above the threshold to 6 mA. (**C**) DC-current induced IMT. The red and blue curves correspond to the increase and decrease of current, respectively. The heating branch is used to simulate the current-density distribution when increasing current, whereas the case for decreasing current is simulated with cooling branch. Thus, the thermal hysteresis may lead to the large differences in the critical currents, assisted by the distinction in the initial state while stimulation. (**D**) Schematic of the calculated magnetic field in the NV-center layer, which is the integration of magnetic field

induced by current density in the VO_2 film. (**E**) Cross-sectional plot of the current density along the dashed line in (B). (**F**) The normalized magnetic-field intensity when setting the space of the NV-center layer and the VO_2 layer to 1-4 μm, respectively. The dots show the normalized intensity of modulated PL in the measurement, which is positively correlated with the magnetic field. Thus, the space of the NV-center layer and the VO_2 film in this case is about 1-2 μm.

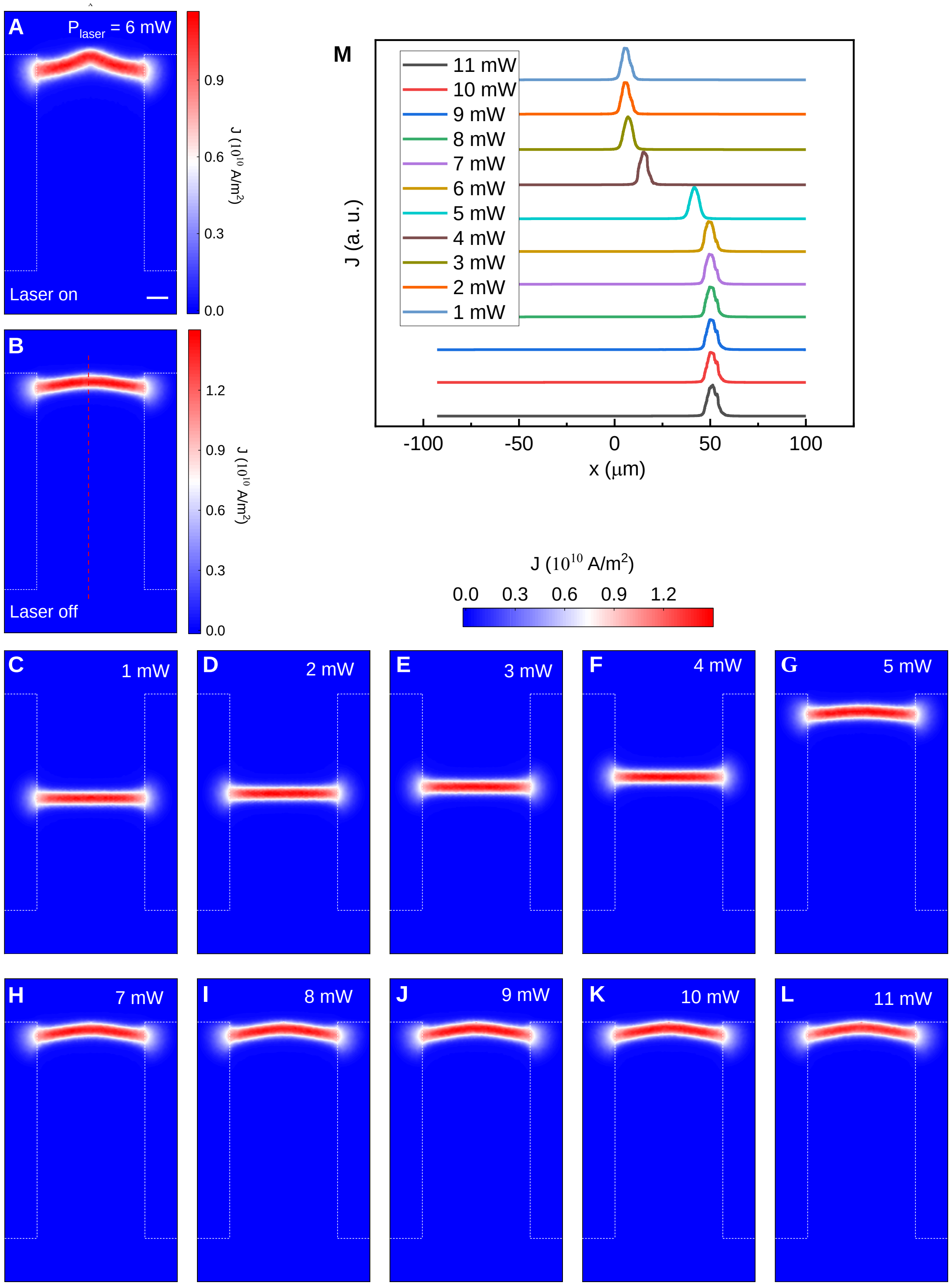

Fig. S8. Filament location controlled by the power of the focused laser in a simulation with 10-mA current. The base temperature in this simulation is 332 K. (**A**) Distribution of current density displays that the filament forms at the edge of electrodes when the laser is focused to x =50 μm and the power is set to 6 mW. The scale bar is 10 μm. (**B**) Current density distribution when the laser is removed shows that the filament is stabilized at x =50 μm, which is simulated

using (A) as the initial state. (**C-I**) Current density distribution when the laser is focused to x =50 µm and sequentially removed. The power is set to 1-11 mW, respectively. (**M**) Current density along the dashed line in (B) with different laser powers, showing a jump in filament location similar to the experimental results in the main text.

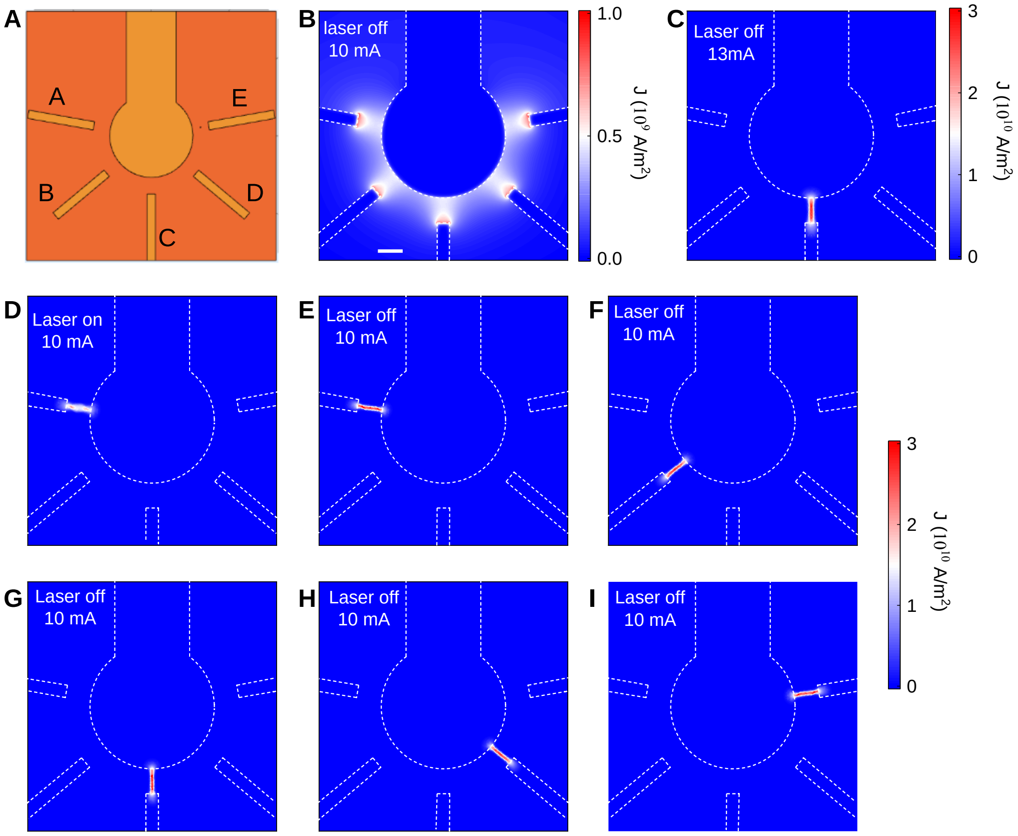

Fig. S9. Simulation of the artificial neuron. (**A**) Geometry of experimental device of Fig. 4 in the main text, including 100-nm thick VO_2 film, 200-µm thick Al_2O_3 substrate and 100-nm thick grounded gold electrodes, labeled with A, B, C, D, and E, respectively. The base temperature in the simulation is 332 K. (**B**) The dispersed current-density distribution when a 10-mA current is applied on the common electrode. The scale bar is 20 µm. (**C**) The filament forms to connect the electrode C when the current is increased to 13 mA, which is caused by a small asymmetry in the modeling process. (**D**) The current density distribution shows that a filament forms in channel A when the 10-mW laser is focused on channel A by applying the heat flow to a 1-µm diameter disc in the middle of the selected channel. The current is subsequently increased to 10 mA. (**E**) Filament stabilizes in channel A when the laser is removed. (**F-I**) Current density distribution when focusing a 10-mW laser on channel B, C, D, and E, triggered by current and removing the laser sequentially, respectively.

S4. The verification for control of filament location by focused laser

We have tested another device on a different sample and observed the phenomena similar to the device in the main text. The resistance-temperature curve reveals the IMT of the VO_2 film, as shown in Fig. S10A. In the following experiments, the base temperature is kept stable at 332 K. The field-induced IMT occurs when the current reaches the threshold of 3.5 mA, which is lower

than the critical current of the device in the main text. However, the filament requires more current to reach the steady state, which is 6 mA. The difference between the devices may be caused by defects in the VO_2 films. The high density of defects between the electrodes may cause the resistance to drop to a new metastable state. In the absence of heating laser, the conducting filaments forms at approximately x = -10 μm instead of the center of device in main text, revealing the apparent effect of defects, as shown in Fig. S10D. The pattern of conductive filaments is heavily influenced by the resistivity change across the transition (*16*). The imaging results are also governed by the distance of NV-center layer and VO_2 film, as illustrated in Fig. S7.

Following this, we investigate the filament formation location as a function of laser position and power. The focused laser is sequentially positioned to x = -50, -25, 0, 25, and 50 μm, respectively, and the laser power is adjusted with the same procedure in the main text. After the field-induced filament generation, normalized PL modulation intensity is measured along the midline between the electrodes. In the test, the DC current used to stabilize the filament is set to 4 mA. Figure S11 illustrates the filament locations as a function of laser power. There is the hysteresis on heating-laser power according to the experiment, comparable to the phenomena described in the main text. Figure S11 also shows the filament imaging for 18-mW laser power case with different laser positions.

As shown in Fig. S12, the resistive switching process during electric field-induced IMT reveals the nature of short- and long-term memory in VO_2 films, which lays the foundation for the implementation of dynamic network structure based on the memristors. State 1 is the initial state when the device is heated to 332 K. The current is then increased above the threshold to trigger the IMT, resulting in state 2 with a conducting filament. After that, the current is reduced to 0.2 mA, and the resistance is monitored over time. The resistance increases rapidly over few tens of seconds and then gradually over the next few hours, which is state 3. We repeat the switch between states 2 and state 3 and observe similar results, as shown in Fig. S12B. The slowly changing state 3 does have lower resistance than state 1, despite the same temperature and test current. Therefore, the change in resistance between states 1 and state 3 should be attributed to the thermal hysteresis of the VO_2 film, with the slowly increasing resistance suggesting a gradual structure transition from the conducting rutile phase to the insulating monoclinic phase for percolation (*48*). The thermal hysteresis could produce a decrease in resistivity in the previous filament region, resulting in a lag in the filament formation location depending on the laser power. In this way, the thermal hysteresis can be utilized to generate the long-term nonvolatile memory.

The sharp increase in resistance should be attributed to rapid heat dissipation during the first tens of seconds when switching from state 2 to state 3, as shown in Fig. S12C. Due to the inhomogeneous current distribution, the local temperature in the filament region is considerably higher in state 2 than in other regions. When the current is removed, the Joule heat, which has very little energy in the filament region, dissipates rapidly, resulting in a sharp rise in resistance, so that the heat dissipation can be used to create a short-term volatile memory.

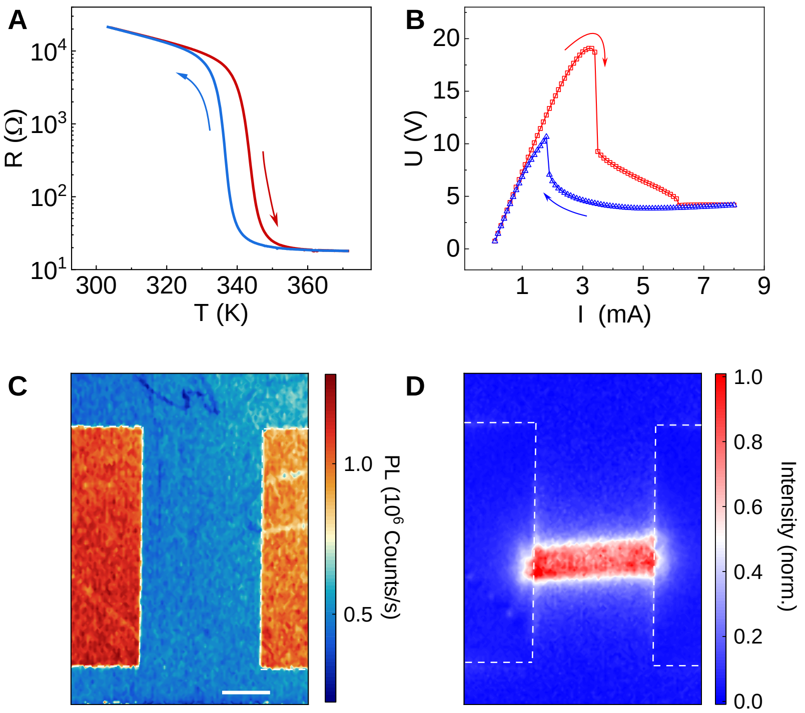

Fig. S10. Temperature and electric-field triggered IMT and conducting filament imaged in another device. (**A**) Thermally triggered IMT, accompanied by orders of magnitude decrease in resistance. The red and blue curves correspond to the heating and cooling branches, respectively. (**B**) DC-current induced IMT. The red and blue curves correspond to the increase and decrease of current, respectively. (**C**) Confocal PL image of the NV centers positioned on the VO_2 device with a 500-nm resolution. The PL on the electrodes is substantially higher than that on the VO_2 film. The scale bar is 20 μm. (**D**) Direct imaging of the current density distribution between the electrodes by scanning the PL-modulation intensity at 4 mA. The electrodes are framed by white dashed lines.

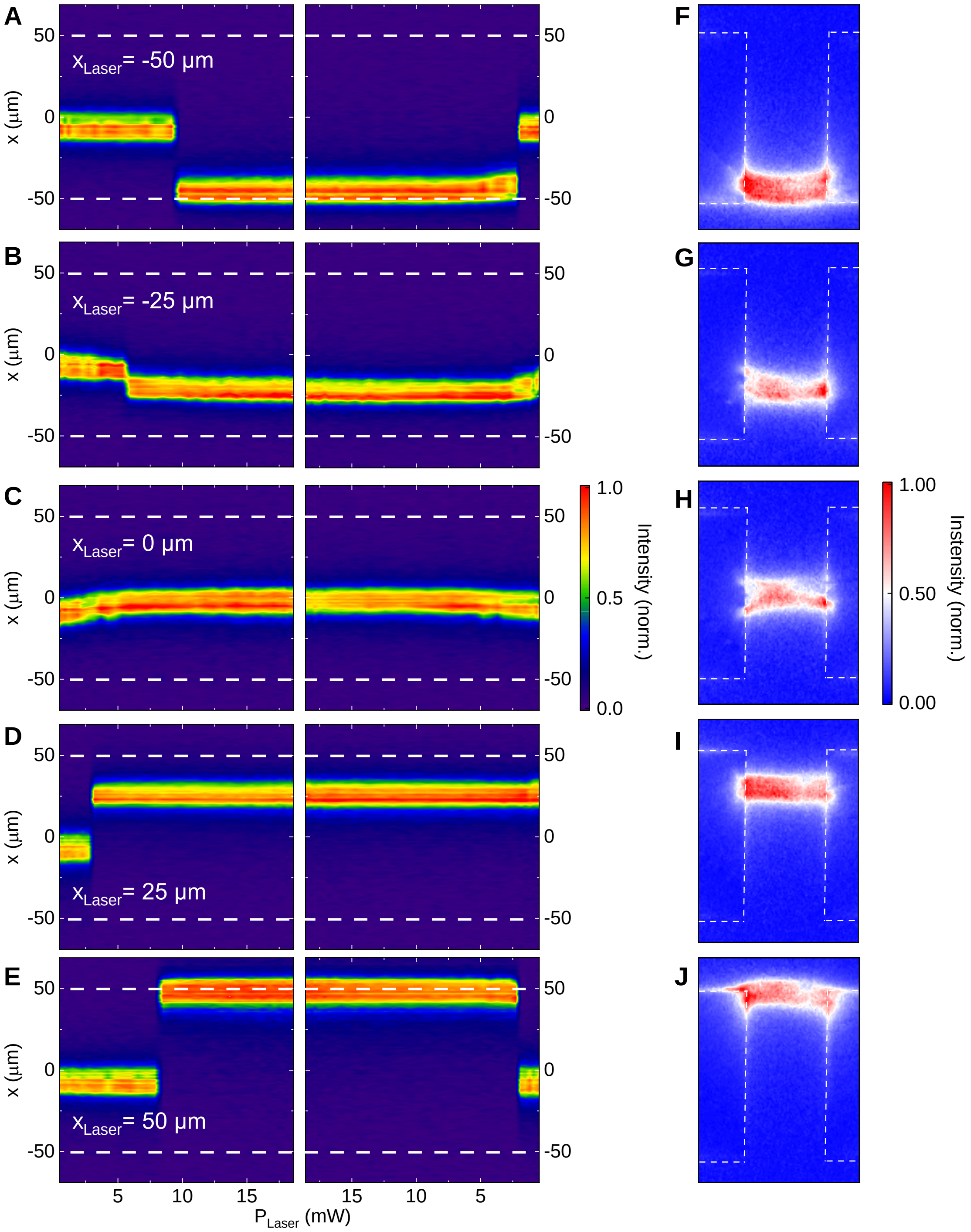

Fig. S11. Control of filament location by focused laser. (**A-E**) The intensity distribution when scanning in the direction perpendicular to the current along the midline between the electrodes. The focused-laser position is set to -50, -25, 0, 25, and 50 μm, respectively, with the adjustable power as the operation of Fig. 3F in the main text. The filament location exhibits a jump similar to the phenomena in the main text. The white dashed lines mark the boundaries of the electrode pair in the VO_2 device. (**F-J**) Imaging of the filament when the focused laser is set to a maximal power of 18 mW and focused at each corresponding position.

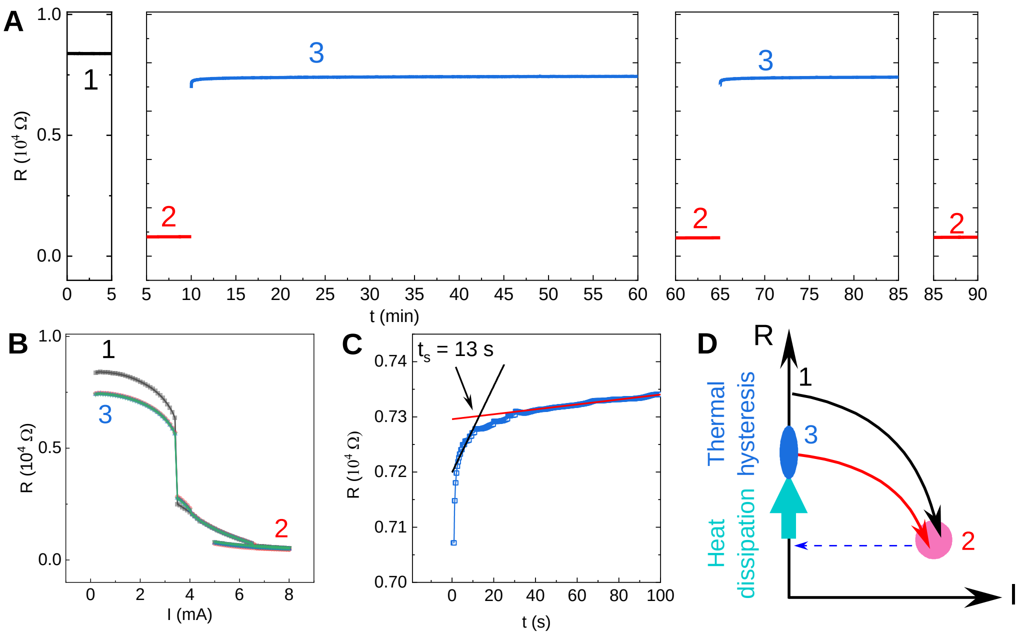

Fig. S12. The changes in resistance at different states of two-terminal VO_2 device reveals the nature of short-term and long-term memory. (**A**) State 1 is the initial state when the device is heated to 332 K, and then the current is increased above the threshold to trigger IMT, resulting in state 2, where IMT induces a conducting filament. Afterwards, the current is reduced to 0.2 mA and state 3 returns to a high resistance for a short time and remains a lower resistance than state 1 for a long time. There is also a gradual increase in the subsequent hours for state 3, which suggests a gradual transition of the structure from a conducting rutile phase to an insulating monoclinic phase. We repeat the switch between states 2 and 3 and observe a similar phenomenon. (**B**) Current-induced IMT. Black line shows the original transition from state 1 to 2, and others show a repeatable transition from state 3 to 2. (**C**) Transition from state 2 to 3 shows a sharp increase in resistance in the first few tens of seconds, and this short-term change may be attributed to the dissipation of Joule heat. (**D**) Summary for the transitions between states. After removing current in state 2, the resistance of the device will rise rapidly due to the heat dissipation, and then increase slowly due to the percolation effect with some residual metallic regions converting to insulator state gradually around the filament, which can bring about thermal hysteresis.

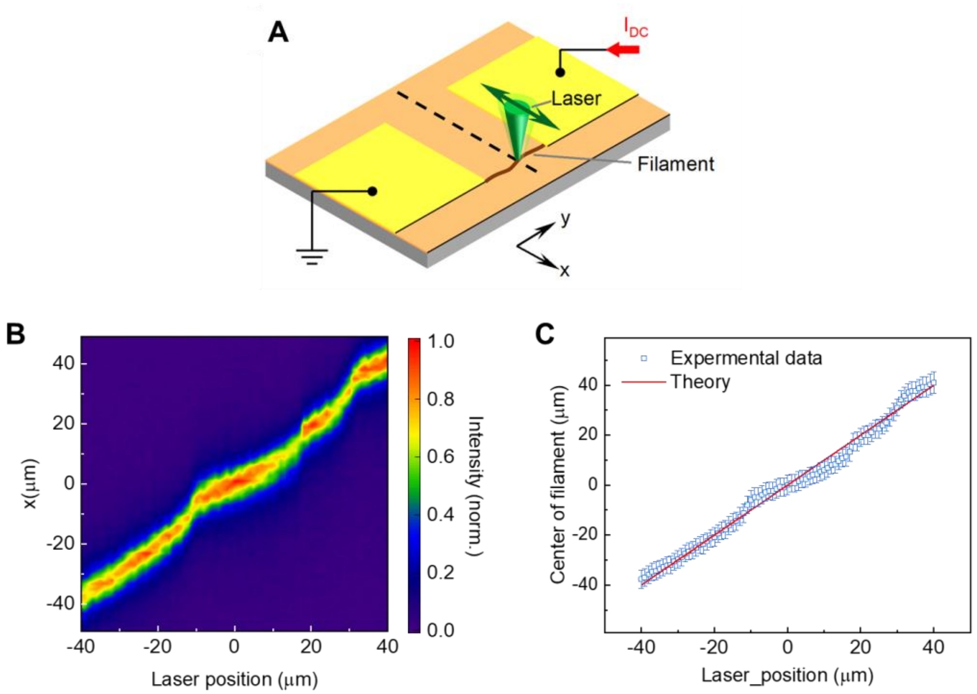

Fig. S13. The movement of conducting filament in response to the position of laser spot. (**A**) Operating procedure for measuring the filament location. The laser is focused on the dashed line on the VO_2 device with varying position on x axis (the laser power is fixed at 13 mW for enough local heat), and the filament is triggered by a DC current before being detected by NV centers. Normalized PL-modulation intensity is also scanned along the dashed line, in the direction perpendicular to the current, for detecting the center of conducting filament. (**B**) The intensity in the direction perpendicular to the current for each position of heating laser spot. (**C**) Blue scatters show the experimental results for the center of conducting filament in response to the position of laser spot. In theory, the location of filament follows the laser position, shown as red line. The matching of theory and experimental data suggests that the control of laser heating on filament can be achieved to about 1 μm.
